# Social capital in South Africa’s minibus taxi industry: networks, trust, and challenges

**DOI:** 10.3389/fsoc.2026.1695191

**Published:** 2026-02-11

**Authors:** Anesipho Klaas-Fobosi, Siyabulela Christopher Fobosi

**Affiliations:** 1 Faculty of Management and Commerce, University of Fort Hare, Alice, South Africa; 2 Faculty of Law, Department of Public and Constitutional Law, University of Fort Hare, Alice, South Africa

**Keywords:** informal economy, minibus taxi industry, networks, public transport, regulation, social capital

## Abstract

**Introduction:**

The minibus taxi industry is a cornerstone of South Africa’s public transport system, providing daily mobility to millions of commuters, particularly in low-income and peri-urban areas. Despite its economic and social importance, the industry remains largely informal, characterized by precarious labor conditions, weak regulatory oversight, and recurring conflict among operators. This paper examines how social capital – understood as networks, trust, and shared norms – shapes governance, labor relations, and operational practices within the industry.

**Methods:**

This study employs a qualitative thematic literature review to synthesize existing research, empirical findings, and policy debates on social capital in South Africa’s minibus taxi industry. A corpus of sources published between 2000 and 2025 was identified through academic databases and institutional repositories. After screening for analytical relevance, 62 sources were retained for full thematic analysis using iterative reading and manual coding to identify recurring concepts and patterns.

**Results:**

The analysis highlights the dual nature of social capital: while dense internal networks (bonding social capital) enable coordination, route allocation, and business continuity, they also reinforce power asymmetries, exclusion, and labor precarity, especially for drivers. The review further shows how gaps between informal governance structures and formal state regulation (linking social capital) undermine efforts at industry reform. Trust-based networks facilitate essential operations and informal financial resilience, yet the absence of legal oversight often leads to exploitative labor practices and violent confrontations over lucrative routes.

**Discussion:**

The paper concludes that bridging the gap between existing informal governance and formal institutions is vital for industry stability. It proposes policy interventions that leverage existing social capital while strengthening formal governance, labor protections, and financial inclusion. Recommendations include formalizing taxi associations as cooperative enterprises to access government subsidies and secure legal recognition, implementing standardized employment contracts, and adopting digital technologies for fare collection to improve transparency and reduce conflict.

## 1 Introduction

The minibus taxi industry in South Africa is a vital component of the country’s public transport system, providing mobility to millions of commuters, particularly in low-income communities where formal public transport options are limited or unreliable. The industry emerged in response to the need for affordable and accessible transportation, especially for historically marginalised populations. Despite its significance in facilitating economic activity and social mobility, the minibus taxi sector remains largely informal, operating within a complex and self-regulated network of associations, drivers, and operators (Fobosi, 2021; Adler and Kwon, 2002). While this informality allows for flexibility and adaptability, it also brings numerous challenges, including precarious working conditions (Barchiesi, 2011), regulatory conflicts, and instances of violence. As one of the dominant transport modes in South Africa, minibus taxis account for approximately 70% of daily public transport commutes, far exceeding the usage of buses and trains (Fobosi, 2019). However, the industry’s reliance on informal structures means that it lacks many of the legal protections and organisational efficiencies associated with formalised transport systems. The absence of standardised labour regulations results in widespread job insecurity, poor working conditions, and disputes over wages (Barchiesi, 2008). Additionally, the industry’s self-regulated nature often leads to competition over routes, occasionally culminating in violent confrontations among taxi associations. These challenges underscore the need to better understand the structural dynamics of the industry and the role of social capital in shaping its operations.

The minibus taxi industry comprises three primary actors:

- Taxi associations: Informal collectives of taxi owners and operators that regulate routes, fares, and disputes.
- Taxi owners: Individuals or groups who own vehicles and lease them to drivers.
- Drivers: Typically non-owner operators who drive taxis and work long hours under precarious conditions.

While associations dominate decision-making, drivers bear the brunt of financial and labour instability. This paper examines how social capital shapes interactions among these actors, with a focus on power imbalances and collective action. Minibus taxis primarily serve short-to medium-distance urban routes (e.g., 10–50 km), charging fares 30–50% lower than formal alternatives like buses (Fobosi, 2019). Over 250,000 taxis operate nationally, organised into 1,500 associations (Fobosi, 2021). Drivers typically lease vehicles from owners for ZAR 300–500 daily, necessitating 12 + hour shifts to break even. This informal leasing system (Barrett, 2003), devoid of contracts, exacerbates drivers’ vulnerability to exploitation. This paper adopts a thematic review methodology to synthesise existing literature, empirical findings, and policy debates on social capital in South Africa’s minibus taxi industry.

## 2 Methodology: thematic literature review

This study employs a qualitative thematic literature review to synthesise existing research on social capital in South Africa’s minibus taxi industry. A thematic review is appropriate given the fragmented nature of the literature and the study’s aim to integrate empirical findings, policy analyses, and theoretical debates rather than generate new primary data.

The literature search was conducted between March and June 2025 using academic databases including Google Scholar, Scopus, Web of Science, and Sabinet, as well as institutional repositories for South African theses and policy reports. Search terms included combinations of *“minibus taxi industry,” “informal transport,” “social capital,” “informal economy,” “taxi associations,”* and *“precarious labour.”* The search focused on English-language sources published between 2000 and 2025, reflecting both foundational and contemporary contributions.

An initial corpus of approximately 110 sources was identified. These were screened based on relevance to (a) informal transport or informal economies, (b) social capital or informal governance, and (c) empirical or conceptual engagement with the South African taxi industry or comparable Global South contexts. After removing duplicates and sources with limited analytical relevance, 62 sources were retained for full thematic analysis.

Included sources comprised peer-reviewed journal articles, academic theses, policy reports, and selected working papers. Opinion pieces, newspaper articles, and purely descriptive accounts without analytical engagement were excluded. Priority was given to studies with clearly articulated methods, whether qualitative, quantitative, or mixed-methods. The selected literature was subjected to iterative reading and manual coding to identify recurring concepts, patterns, and tensions. Themes were derived inductively and refined through analytic comparison across sources. The final thematic structure focuses on (i) informal regulation and governance, (ii) trust-based networks and labour relations, and (iii) challenges and contradictions of social capital, particularly violence, exclusion, and precarity.

Rather than assessing statistical representativeness, the review evaluates the *analytical contribution* and contextual relevance of studies. The synthesis aims to link theory and evidence by explicitly grounding claims in identifiable strands of the literature while acknowledging interpretive extrapolation where applicable.

## 3 Understanding social capital in informal economies

### 3.1 Conceptual framework: social capital in the minibus taxi industry

This paper adopts a multidimensional conceptualisation of social capital, drawing on Woolcock’s distinction between bonding, bridging, and linking social capital. Within the minibus taxi industry, these forms operate simultaneously but unevenly across actors.

Bonding social capital refers to dense, inward-looking networks among taxi owners and associations, which facilitate coordination, mutual protection, and enforcement of informal rules. Bridging social capital captures horizontal relationships between different associations, drivers, and allied informal actors, enabling limited cooperation across organisational boundaries. Linking social capital denotes vertical connections between the industry and formal institutions such as government departments, financial institutions, and law enforcement agencies—connections that remain weak and contested.

**Figure 1:**
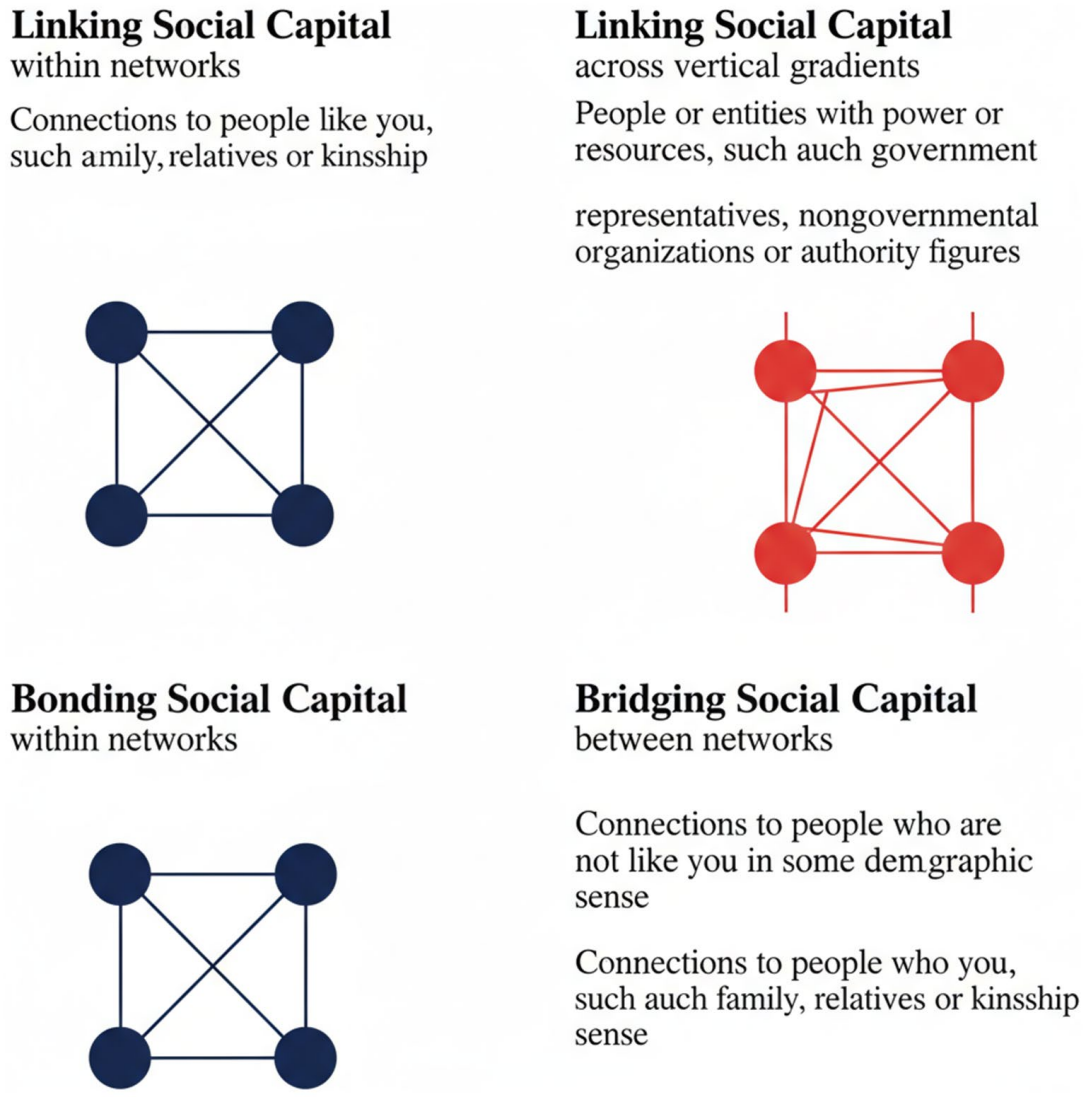
Conceptual framework of bonding, bridging, and linking social capital in the minibus taxi industry.

Figure 1 summarises this framework and illustrates how different forms of social capital shape governance, labour relations, and conflict within the industry. Importantly, the framework foregrounds power asymmetries by showing how bonding social capital can simultaneously enable collective action and reproduce exclusion and precarity, particularly for drivers.

Social capital is a critical concept in understanding the minibus taxi industry, as it explains the ways in which relationships, trust, and networks contribute to the industry’s functioning. Broadly defined, social capital refers to the networks, shared norms, and trust that enable collective action within a society or economic sector (Putnam, 1993). In the context of informal economies, where formal institutions and regulations are weak or absent, social capital plays a fundamental role in sustaining economic activities and ensuring cooperation among stakeholders (Klaas, 2020; Fobosi, 2021; Woolcock and Narayan, 2000). While Putnam’s concept of social capital emphasises networks, trust, and norms as mechanisms for collective action, Bourdieu’s perspective introduces a critical dimension of power and conflict. Bourdieu defines social capital as ‘the sum of resources, actual or virtual, that accrue to an individual or group by virtue of possessing a durable network of more or less institutionalised relationships of mutual acquaintance and recognition’ (Bourdieu, 1986). In the context of the minibus taxi industry, this perspective helps explain how taxi associations use social capital to maintain control over lucrative routes and enforce informal regulations, often leading to violent conflicts. Additionally, Bourdieu’s concept of symbolic capital – the prestige and legitimacy conferred by social networks – sheds light on how associations gain authority and recognition within the industry, even in the absence of formal institutional support. Bourdieu’s (1986) framework is particularly salient here: social capital is not merely a cooperative resource but a tool for maintaining dominance. Taxi associations leverage networks to monopolise routes, while drivers—lacking equivalent capital—face exploitation. This power asymmetry underscores the duality of social capital: it fosters cohesion among elites but perpetuates precarity for marginalised actors (Bourdieu and Wacquant, 1992).

Recent studies (Khuzwayo, 2023; Kerzhner, 2023) confirm social capital’s dual role in informal transport sectors globally, while South African-specific analyses (Fobosi, 2021; Klaas, 2020) detail its localised manifestations. This paper synthesises such contemporary evidence to advance the debate. Within the minibus taxi industry, social capital manifests through bonding social capital, bridging social capital, and linking social capital (Woolcock, 1998). Bonding social capital exists within tightly knit networks, such as taxi associations and driver collectives, where mutual support and trust create a sense of belonging. Bridging social capital, on the other hand, facilitates cooperation between different groups within the industry, such as taxi operators, regulatory bodies, and local businesses (Klaas, 2020). Linking social capital refers to the relationships between the taxi industry and external institutions, such as government agencies, financial service providers, and law enforcement authorities. These forms of social capital influence how resources, information, and opportunities are distributed within the sector.

## 4 The informal regulatory framework and its implications

One of the defining features of the minibus taxi industry is its informal regulatory framework, which operates outside the traditional governance structures that oversee public transport systems. While the government has attempted to regulate the industry through initiatives like the Taxi Recapitalisation Programme (TRP), implementation has been inconsistent, with limited success in formalising operations (Fobosi, 2021; Granovetter, 1973). Instead, taxi associations act as self-regulating entities, controlling route allocations, fare structures, and conflict resolution mechanisms.

Taxi associations, though informal, serve as powerful institutions that mediate disputes and enforce industry norms. Membership in these associations provides operators with a sense of security and access to shared resources. However, because these associations function outside formal legal frameworks, conflicts often escalate into violent confrontations, particularly over lucrative routes. The reliance on informal agreements rather than legally binding contracts exacerbates tensions, making it difficult to establish long-term stability within the sector.

## 5 The role of trust and networks in the minibus taxi industry

Trust is a crucial element of social capital in the minibus taxi industry. Drivers, operators, and associations rely on trust-based relationships to conduct daily operations. This is evident in how taxi owners entrust their vehicles to drivers, often without formal employment contracts. Drivers, in turn, must trust that they will earn sufficient income through a daily cash-based system, which is subject to fluctuations in commuter demand and competition. Moreover, the industry’s network-based structure ensures that operators and drivers can access essential resources, such as vehicle financing and maintenance services. Many taxi owners depend on rotating credit schemes and informal lending networks to purchase and maintain their vehicles, as traditional financial institutions often view the industry as high-risk (Fukuyama, 1995; Lin et al., 2001). These financial arrangements underscore the importance of social capital in sustaining business operations in an environment with limited access to formal banking services. While these networks provide a degree of resilience, they also pose risks. The absence of legal oversight means that financial disputes are settled informally, sometimes leading to exploitative practices. In some cases, taxi owners force drivers to work long hours under strenuous conditions to meet daily payment requirements. The lack of standardised labour protections means that drivers have little recourse in cases of unfair treatment, further exacerbating precarious working conditions (Berrones-Sanz, 2014).

## 6 Challenges to social capital in the minibus taxi industry

While social capital underpins the daily functioning of the minibus taxi industry, it is also a source of instability and exclusion. The most visible manifestation of this contradiction is recurrent violence linked to route competition and territorial control. Empirical studies document how taxi associations deploy dense social networks to defend market share and enforce informal authority, often in the absence of effective state mediation (Fobosi, 2019, 2021). These conflicts are not incidental but structurally embedded in an industry where access to routes determines economic survival. As a result, bonding social capital strengthens internal solidarity while undermining broader trust and cooperation across the sector. Survey-based and qualitative studies indicate that a majority of drivers work shifts exceeding twelve hours per day to meet daily lease payments, particularly in urban routes with high competition (Fobosi, 2021; Khuzwayo, 2023). Government attempts to formalise industry have been met with resistance from associations that fear losing autonomy. The challenge lies in balancing regulatory oversight with the existing informal governance structures. Without mechanisms to bridge social capital between taxi operators and formal institutions, efforts to integrate the industry into national transport policies will likely face continued opposition. Another pressing issue is the precarious employment conditions for drivers. Unlike workers in formal industries, taxi drivers operate without employment contracts, medical benefits, or retirement plans. The informal agreements that sustain the industry offer flexibility but also expose workers to financial instability. Addressing this challenge requires policy interventions that acknowledge the informal structures while promoting fair labour practices.

## 7 Toward a sustainable minibus taxi industry

Given its significance in South Africa’s transport landscape, the minibus taxi industry requires a multi-stakeholder approach to enhance its stability and efficiency. Strengthening bridging and linking social capital can facilitate collaboration between taxi associations, government agencies, and financial institutions. Establishing formal platforms for dialogue between stakeholders could help address issues such as violence, financial exclusion, and labour rights. The prevalence of violence in the minibus taxi industry can be understood through Bourdieu’s lens of social capital as a tool for maintaining power. Taxi associations, as dominant groups, use their social networks to control lucrative routes and exclude competitors, often resorting to violent means to enforce their authority. This dynamic reflects Bourdieu’s concept of symbolic violence, where dominant groups impose their norms and values on others, reinforcing their control without the need for formal institutional backing. The lack of formal governance structures exacerbates these power struggles, as associations rely on informal networks to maintain their dominance, often at the expense of drivers’ and commuters’ well-being.

One potential solution is the formalisation of taxi associations as cooperative enterprises, which would enable them to access government subsidies, financial services, and training programmes (Coleman, 1988; Burt, 1992; Fobosi, 2021). This approach could also facilitate the development of standardised employment contracts for drivers, ensuring greater financial security and labour protections. Formalising taxi associations would not only provide them with legal recognition but also convert their informal social capital into institutionalised capital, as described by Bourdieu. Institutionalised capital refers to formal recognition and legitimacy, such as educational qualifications or legal status, which can enhance an organisation’s access to resources and authority. By formalising associations, the industry could reduce its reliance on informal networks and violent enforcement mechanisms, shifting the balance of power toward more transparent and accountable governance structures. This would also enable associations to access government subsidies, financial services, and training programmes, further stabilising the industry.

Furthermore, investing in digital technologies to streamline fare collection, route management, and dispute resolution could reduce conflicts and improve operational efficiency. Mobile payment systems, for instance, could enhance transparency in financial transactions, reducing reliance on cash-based economies that are susceptible to exploitation.

## 8 Recommendations

To leverage social capital for the improvement of the minibus taxi industry, a multi-faceted approach is required, addressing formalisation, capacity building, trust, financial inclusion, and community engagement. These recommendations aim to strengthen the industry’s sustainability, efficiency, and overall contribution to South Africa’s transport sector while acknowledging the informal nature of its operations. One of the key challenges in the minibus taxi industry is the lack of formal governance structures, which often leads to inefficiencies, conflicts, and violent disputes over routes. Encouraging the formalisation of taxi associations would provide a more structured framework for cooperation and conflict resolution. By registering as formal business entities or cooperatives, taxi associations could gain access to government support, funding, and regulatory benefits while improving internal management (Putnam, 2000; Woolcock, 1998; Fobosi, 2021). This would also enable the government to enforce labour protections, introduce standardised employment contracts, and facilitate negotiations between associations and regulatory bodies. Establishing clear governance structures within associations, such as electing leadership, maintaining financial records, and implementing transparent decision-making processes, would help reduce internal disputes and promote accountability. Furthermore, formalised associations could work alongside government agencies to ensure better route allocation, standardised pricing, and more effective dispute resolution mechanisms, reducing the prevalence of violent conflicts.

Another critical area for intervention is capacity building for taxi operators and drivers. Many taxi operators enter the industry without formal training in business management, financial planning, or conflict resolution. Implementing structured training programmes could enhance their ability to manage finances, handle customer relations, and navigate disputes peacefully. Capacity-building initiatives should focus on financial literacy, helping taxi owners understand budgeting, savings, and loan management to prevent financial exploitation. Additionally, customer service training could improve commuter experiences and encourage trust between passengers and operators. Training on conflict resolution and negotiation skills is also essential, given the industry’s history of violent disputes. By equipping operators and drivers with these skills, associations can foster a more professional industry, ensuring stability and long-term growth.

Trust is a fundamental element of social capital in the minibus taxi industry, yet it remains fragile due to inconsistent fare structures, security concerns, and disputes between stakeholders. Strengthening trust within the industry requires transparent and standardised operational procedures that benefit all participants. One way to achieve this is through the introduction of standardised fare systems, ensuring that passengers are not exploited and that drivers receive fair compensation. Currently, fare-setting is often arbitrary, leading to frequent disputes between operators and commuters. Establishing a regulated fare structure – possibly incorporating digital payment systems – could improve transparency and reduce conflicts. In addition to fare standardisation, improving safety measures is crucial. Many commuters hesitate to use minibus taxis due to concerns over reckless driving, vehicle safety, and crime. Introducing driver certification programmes, mandatory vehicle inspections, and surveillance systems in taxis could enhance safety and boost public confidence. Furthermore, the establishment of grievance mechanisms, such as dedicated complaint hotlines or industry ombudsmen, could provide passengers and drivers with a formal channel to report issues, ultimately fostering greater accountability and trust.

A significant challenge for many taxi operators and drivers is their limited access to formal financial services, which forces them to rely on informal lending networks that often charge exorbitant interest rates. Facilitating access to microloans, savings accounts, and insurance products could significantly improve financial security for industry participants. Many financial institutions are reluctant to extend credit to taxi operators due to perceived risks and a lack of collateral. However, by working with formalised taxi associations, banks and microfinance institutions could develop tailored financial products that meet the needs of the industry. Providing low-interest microloans for vehicle purchases or maintenance would help operators invest in safer, more fuel-efficient taxis. Additionally, offering savings accounts and pension plans could ensure financial stability for drivers, many of whom currently lack retirement security. Another crucial financial service is insurance, which remains largely absent in the industry. Many taxi owners and drivers do not have access to vehicle, health, or life insurance, leaving them vulnerable to financial ruin in the event of accidents or illness. Collabourating with insurance providers to develop affordable, industry-specific insurance plans could mitigate risks and improve economic resilience.

Finally, community engagement is vital in fostering stronger relationships between the minibus taxi industry and the communities it serves. Taxi operators and drivers are deeply embedded in the communities they transport, yet interactions are often transactional rather than collabourative. Establishing community advisory boards where passengers, local business owners, and taxi operators can discuss service improvements, safety concerns, and commuter needs could create a more inclusive transport system. Additionally, launching public awareness campaigns that educate commuters about their rights, fare structures, and available grievance mechanisms could reduce tensions between passengers and drivers. Community outreach programmes that involve taxi operators in local development initiatives, such as youth mentorship or road safety education, could further strengthen trust and cooperation. By aligning industry interests with community well-being, operators and passengers can work together toward a more sustainable and passenger-friendly transport sector.

The improvement of the minibus taxi industry requires a holistic strategy that strengthens governance structures, enhances skills development, builds trust, expands financial access, and fosters community engagement. Formalising taxi associations would provide stability and accountability, while capacity-building initiatives would ensure that operators and drivers develop the skills needed to run sustainable businesses. Strengthening trust through standardised fares, improved safety measures, and transparent grievance mechanisms would enhance commuter confidence and industry reputation. Expanding access to formal financial services would provide greater economic security, reducing reliance on informal lending systems that exploit drivers and owners. Finally, fostering community partnerships would align the interests of the taxi industry with those of local commuters, creating a transport system that is not only functional but also socially inclusive. Implementing these recommendations could transform the minibus taxi industry into a more efficient, equitable, and sustainable component of South Africa’s public transport infrastructure.

To address the power imbalances within the minibus taxi industry, formalisation efforts should aim to redistribute power, reducing the dominance of associations and empowering drivers and commuters. This could involve creating independent regulatory bodies that include representatives from all stakeholders, ensuring a more equitable distribution of resources and decision-making authority. Additionally, initiatives to build symbolic capital for drivers and commuters – such as public awareness campaigns that highlight their contributions to the industry and the broader community – could enhance their legitimacy and influence, fostering a more inclusive and sustainable transport system.

## 9 Discussion and conclusion

### 9.1 Contributions to theory and policy

This review contributes to social capital theory by demonstrating how classic frameworks require modification when applied to informal transport labour. Rather than treating social capital primarily as a collective good, the synthesis foregrounds its role in reproducing power and precarity within informal governance systems. Empirically, the paper advances existing South African scholarship by integrating labour, governance, and transport literatures into a single analytical frame. From a policy perspective, the study shows that reform efforts that ignore embedded social networks are likely to fail, underscoring the need for hybrid governance approaches that combine formal regulation with existing informal institutions.

This thematic review has systematically examined the complex interplay between social capital and South Africa’s minibus taxi industry, revealing how informal networks simultaneously sustain and undermine the sector. The analysis demonstrates that while bonding social capital within taxi associations facilitates daily operations and conflict resolution (Putnam, 2000), these same networks reinforce power asymmetries through Bourdieu (1986) concept of symbolic capital. Associations leverage their social capital to control lucrative routes, often at the expense of drivers who face precarious working conditions and financial exploitation. This duality lies at the heart of the industry’s challenges, where organic social structures both enable survival and perpetuate instability. Where the literature does not provide precise quantitative estimates, the analysis draws interpretively on recurring qualitative patterns reported across multiple studies.

The review identifies three critical areas requiring attention. First, the persistent formalisation paradox emerges clearly - government interventions like the Taxi Recapitalisation Programme have achieved limited success because they fail to engage with existing social architectures. Second, the driver-owner relationship, built on informal agreements, systematically disadvantages drivers, with recent studies indicating 60–75% work more than twelve-hour days to meet payment obligations (Fobosi, 2021; Khuzwayo, 2023). Third, the absence of bridging capital between the industry and formal institutions exacerbates financial exclusion and violent competition for routes.

These findings carry significant theoretical and practical implications. Theoretically, they extend social capital frameworks by demonstrating their contextual limitations in informal economies - what functions as collective action for some becomes a tool of exclusion for others. Practically, they suggest three intervention pathways: gradual formalisation that preserves beneficial networks while introducing accountability mechanisms; hybrid financial products that combine traditional stokvel models with formal banking services; and digital mediation through technologies like blockchain for transparent route allocation. Each approach recognises social capital as both an obstacle and an asset in reform efforts.

Several limitations qualify these conclusions. The available literature exhibits urban bias, with limited research on rural operations, and lacks longitudinal data on network evolution. Furthermore, gendered dimensions of social capital accumulation remain understudied, despite women’s growing participation as owners and operators. These gaps present clear opportunities for future research, particularly comparative studies across regions and demographic groups, as well as impact assessments of technological interventions in the sector.

Therefore, this review underscores that meaningful improvement in the minibus taxi industry requires working with, rather than against, its embedded social structures. The discussion has highlighted how social capital operates as a double-edged sword - enabling collective action while reinforcing hierarchies. The conclusion emphasises that sustainable solutions must acknowledge this complexity, balancing formal regulation with respect for organic networks that provide vital economic opportunities. For policymakers, this means developing participatory reform processes; for researchers, it necessitates more nuanced studies of power dynamics within informal networks. Ultimately, the South African minibus taxi industry offers valuable insights into the challenges of governing informal economies worldwide, where social capital remains both the foundation of operation and the barrier to transformation.

Recent comparative studies of informal transport in the Global South reinforce the relevance of these findings beyond South Africa. Research on inland waterway transport workers in Banjarmasin, Indonesia, for example, demonstrates how informal workers’ agency and resilience are shaped by cultural norms and governance gaps, producing outcomes similar to those observed in South Africa’s taxi industry. These studies show that informal governance systems can stabilise livelihoods while simultaneously limiting workers’ bargaining power and access to state protection. Situating the minibus taxi industry within this broader literature highlights how social capital operates as a central mechanism of informal mobility governance across diverse contexts, while also underscoring the importance of context-specific power relations.

### 9.2 Limitations and future research

This review is subject to several limitations. The available literature is heavily urban-focused, with limited insight into rural and small-town taxi operations. Gendered dimensions of ownership, labour, and social capital accumulation remain underexplored, despite increasing female participation in the industry. In addition, the reliance on cross-sectional studies limits understanding of how social networks evolve over time. Future research should prioritise longitudinal and comparative studies across regions and transport modes to deepen understanding of informal mobility governance.

## Data Availability

The datasets presented in this article are not readily available. Requests to access the datasets should be directed to sfobosi@ufh.ac.za.
